# O-RADS combined with contrast-enhanced ultrasound in risk stratification of adnexal masses

**DOI:** 10.1186/s13048-023-01243-w

**Published:** 2023-08-03

**Authors:** Yanyun Shi, Huan Li, Xiuhua Wu, Xiaoqin Li, Min Yang

**Affiliations:** Department of Ultrasonography, The Affiliated Changzhou Second People’s Hospital of Nanjing Medical University, Xinglong Lane, Changzhou, China

**Keywords:** Ultrasound, O-RADS, CEUS, Adnexal Tumor, Ovarian Cancer

## Abstract

**Background:**

Ovarian-Adnexal Reporting and Data System (O-RADS) for ultrasound is a lexicon and risk stratification system that includes all risk categories and relevant management recommendation. It has high sensitivity in diagnosing malignant adnexal tumors, but the specificity is lower.

**Objective:**

To explore the value of O-RADS combined with contrast-enhanced ultrasound (CEUS) in risk stratification of adnexal masses.

**Methods:**

A retrospective study was performed on 85 patients with 100 adnexal masses that preoperatively underwent conventional ultrasound as well as CEUS examination and obtained the postoperative pathological results. The masses were classified into O-RADS2, 3, 4, and 5 by conventional ultrasound. After contrast enhancement, the classification of O-RADS was adjusted according to CEUS imaging features. The O-RADS 2 and 3 lesions with suspected malignant features like irregular blood vessels or internal inhomogeneous hyperenhancement were upgraded to O-RADS 4, and the O-RADS 4 lesions with the above features were upgraded to O-RADS 5. The O-RADS 4 lesions with suspicious benign angiographic features like a regular vessel, interior hypoenhancement or non-enhancement were downgraded to O-RADS 3; the O-RADS 5 lesions with rim ring-enhancement and interior non-enhancement were downgraded to O-RADS 3. The sensitivity, specificity, accuracy, PPV, NPV, and AUC of the two methods were compared, taking pathological results as the gold standard.

**Results:**

The sensitivity, specificity, accuracy, PPV, NPV, and AUC of O-RADS and O-RADS combined with CEUS in the diagnosis of malignant adnexal tumors were 96.6%, 66.2%, 75.0%, 53.8%, 97.9%, 0.910 and 96.6%, 91.5%, 93.0%, 82.4%, 98.5%, 0.962, respectively. The specificity, accuracy, PPV, and AUC of O-RADS combined with CEUS were considerably higher than those of O-RADS (*P* < 0.01). Furthermore, both methods had excellent sensitivity and NPV but there were no significant differences between them(*P* > 0.05).

**Conclusion:**

Combination of O-RADS and CEUS can significantly improve the specificity and PPV in diagnosing malignant adnexal tumors. It seems promising in the clinical application of risk stratification of adnexal masses.

## Introduction

Adnexal masses are very common in females, with ovarian cancer having the highest mortality among gynecological malignancies [1]. Ones an adnexal lesion is found on pelvic imaging, the focus of examination is the evaluation that the lesion is benign or potentially malignant. Malignancy need maximal debulking surgery, while benign tumors perform less aggressive treatment or follow-up [2, 3]. So, accurate differentiation between benign and malignant is essential for clinical management. As the first-line method for evaluating adnexal masses, the ultrasound examination has the advantages of displaying clear images in real-time, convenience, low cost and no radiation [4], but the diagnosis is highly dependent on sonographers, who might have some partiality in the description and diagnosis [5].

In 2020, the American College of Radiology (ACR) formally published the *Ultrasound Guideline of Ovarian-Adnexal Reporting and Data System (O-RADS)*, which combined the image-based classification system and the ADNEX model of IOTA [6]. This guideline developed a standardized lexicon to precisely characterize adnexal masses, reducing the fuzziness of ultrasound reports and improving the ability to evaluate the malignant risk of masses. Furthermore, it comprised risk categories with corresponding risk of malignancy and relevant management recommendation [7, 8]. Studies have shown that O-RADS is more sensitive than other classification or prediction models; however, its specificity is not prominent [7, 9, 10].

Traditional ultrasound could preliminarily determine the nature of the adnexal tumors through observing the structure of masses and detecting the blood flow of lesions. Yet the ultrasound manifestations of the adnexal masses are diverse and complex. In addition, conventional color Doppler sonography specializes in displaying large vascular networks, and it is inferior sensitivity to low-speed blood flow or deeply located vessels due to its inherent defects, such as lack of sound reflection from the red blood cells and low signal-to-noise ratio, which limit the understanding of the actual blood flow perfusion of tumors [11, 12]. The contrast-enhanced ultrasound (CEUS) was performed after giving an intravenous injection of a microbubble contrast agent to enhance the contrast between the lesion and normal tissue. This enabled a higher detection rate of the lesion as well as precise visualization of the microvascularity and hemoperfusion characteristics of the tumors, facilitating the differentiation of benign and malignant lesions. Previous studies have demonstrated the effectiveness of CEUS in distinguishing malignancy from benign adnexal tumors [11–13]. This study explored to taking CEUS features into consideration for O-RADS evaluation, investigating the application value of O-RADS combined with CEUS in the risk stratification of adnexal masses.

## Methods

### Study population

**Fig. 1:**
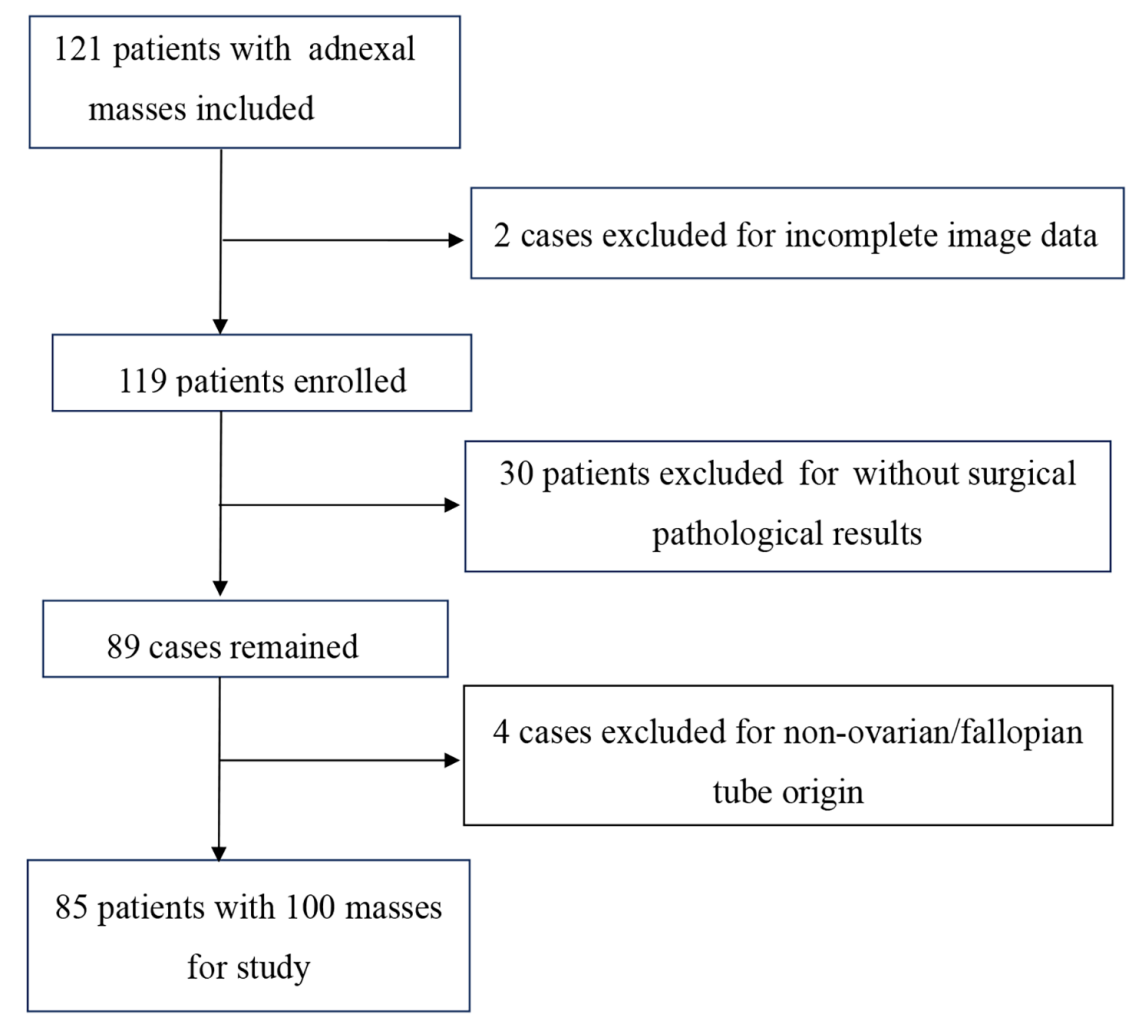
Screening chart of enrolled cases

A total of 121 cases of adnexal masses diagnosed in the Affiliated Changzhou Second People’s Hospital of Nanjing Medical University from September 2020 to May 2022 were collected, and 85 cases were finally enrolled (Fig. 1). Inclusion criteria were the participants who were diagnosed with an adnexal mass and underwent routine ultrasound and CEUS examination before the surgery, and obtained surgical pathological results with complete imaging data. Exclusion criteria were allergic to contrast agents or other allergic contraindications, and the mass was not considered from the ovary or fallopian tube. This study was approved by the ethics committee of Changzhou Second People’s Hospital, and all patients signed the informed consent forms.

### Ultrasound examination

All patients were performed by Philips EpiQ7 ultrasonography device (with a 3 ~ 10 MHz Intracavitary probe; or 1 ~ 5 MHz abdominal probe). Before introducing the contrast agent (SonoVue, Bracco, Italy), 5 mL of saline was injected for dilution, and the mixture was shaken well. Each time, 2.4 mL of the solution was administered via bolus injection, followed by 5 mL of saline flush.

At first conventional ultrasound was carried out. Transvaginal ultrasound was used for small lesions, whereas transvaginal combined with transabdominal ultrasound was used for large lesions. The location, size, maximum diameter, shape, and echogenicity were studied for each lesion. In the case of cystic mass, emphasis was placed on separation, solid components, papillary processes and numbers. For multiple lesions, up to two lesions with the most suspicious malignant lesions were subjected to CEUS. The cross-section covered the whole mass or if the lesion was too large, its solid part, thick cyst wall or separation was selected for the examination. The relationship between the mass and surrounding tissues, swollen lymph nodes or effusion in the pelvic cavity should also be paid attention.

After injecting the contrast agent, time counting was started, the dynamic image was observed continuously for 5 min, and the initial video clip was recorded for at least 120 s. For a case with multiple lesions, the interval between the first and second contrast was more than 15 min. At least one angiogram should show a normal uterus or ovary as a reference. For the consistency of images, CEUS examinations and measurements were performed by the same sonographer with more than 15 years of experience in gynecological ultrasound examination and more than 5 years of experience in CEUS examination. Image analysis was conducted by two experienced sonographers who were blinded to the patients’ clinical data, including pathological findings. A consensus should be made in case of disagreement.

### Image analysis

#### O-RADS

The O-RADS contains six risk assessment categories; O-RADS 0: Technically incomplete evaluation; O-RADS 1: The physiologic category or normal premenopausal ovary; O-RADS 2: Almost certainly benign (malignant risk: < 1%); O-RADS 3: Low-risk lesions (malignant risk: 1 ~ < 10%); O-RADS 4: Moderate-risk lesions (malignant risk: 10 ~ < 50%); O-RADS 5: High-risk lesions (malignant risk: > 50%) [14]. All the enrolled cases are adnexal masses that have been examined, so the subjects of the study are O-RADS 2 ~ 5.

#### O-RADS combined with CEUS

CEUS revealed suspicious benign angiographic features such as regular vessel, late or synchronous wash-in, interior hypoenhancement or non-enhancement, and suspected malignant angiographic features like large and distorted or irregular vessels, early wash-in, inhomogeneous or hyperenhanced internally [11, 13]. The O-RADS classification was modified based on the angiographic features. The O-RADS 2 and 3 lesions with suspicious malignant angiographic features such as an irregular vessel or internal inhomogeneous hyperenhancement were upgraded to O-RADS 4, and the O-RADS 4 lesions with the above features were upgraded to O-RADS 5. The O-RADS 4 lesions with suspicious benign angiographic features like a regular vessel, interior hypoenhancement or non-enhancement were downgraded to O-RADS 3; the O-RADS 5 lesions showing rim ring-enhancement and interior non-enhancement were downgraded to category 3. The classification of O-RADS 2 and 3 lesions with suspected benign angiographic features and O-RADS 5 lesions with suspected malignant angiographic features remained.

### Statistical method

SPSS 26.0 software and MedCalc software(Version19.0.4) were used for statistical analysis. Continuous variables were expressed as means and standard deviations(x ± s), while categorical variables were presented as frequency. The Chi-square test and Fisher’s exact test were used to compare the proportions. In diagnosing adnexal malignancy, Receiver operating characteristic (ROC) curves of O-RADS and O-RADS combined with CEUS were plotted and the optimal cutoff value were identified, respectively. The sensitivity, specificity, accuracy, positive predictive value (PPV), and negative predictive value (NPV) were calculated and compared by Chi-square test. DeLong test was applied to compared the areas under the curves(AUC). *P* < 0.05 was statistically significant.

## Results

### Pathological results

The 85 patients had 100 masses, of which 69 cases were unilateral single lesions, six were unilateral multiple lesions, and ten were bilateral lesions. The patients’ ages ranged from 16 to 86 years old, with an average of (47.9 ± 14.4) years old. There were 56 premenopausal cases and 29 postmenopausal cases. A total of 83 cases were operated at Changzhou Second People’s Hospital, and two cases were operated at other hospitals. Pathological examination confirmed that 71 masses were benign and 29 were malignant (borderline tumors were classified as malignant).

### Conventional ultrasonic examination results

The masses varied from a minimum diameter of 0.8 cm to a maximum of 30 cm, with a mean diameter of (7.8 ± 4.5) cm. The ultrasound revealed 18 unilocular cysts with no solid components, 28 unilocular cysts with solid components, 20 multilocular cysts without solid components, 10 multilocular cysts with solid components, and 24 solid or mostly solid (solid component ≥ 80%). A total of 4 cases with ascites, 3 cases with peritoneal nodules, and 3 cases with enlarged lymph nodes in the pelvis and abdomen were detected.

### O-RADS classification

Among the 100 masses, 19 were classified as O-RADS 2 (19%), 29 were O-RADS 3 (29%), 32 were O-RADS 4 (32%), and 20 were O-RADS 5 (20%) (Table 1). According to the pathological results, the corresponding proportions of malignancy were 0% (0/0), 3.4% (1/29), 31.3% (10/32) and 90% (18/20). There were 24 (33.8%) benign tumors classified as O-RADS 4 or 5, primarily with ovarian endometriosis cysts, mucinous cystadenomas, simple cysts, and fibrotheca cell tumors. 1 (3.4%) malignant tumor was classified as O-RADS 3.

**Table 1:** Classification of 100 adnexal masses by the two methods

Pathological results	Number		O-RADS					O-RADS combined with CEUS			
			2	3	4	5		2	3	4	5
Benign											
Ovarian endometriosis cyst	16		6	3	6	1		6	9	1	
Ovarian serous cystadenoma	8		4	4				4	4		
Ovarian mucinous cystadenoma	9			6	3				8	1	
Ovarian mature teratoma	9		3	5	1			3	6		
Ovarian goiter	5			4		1			4		1
Simple ovarian cyst	8		4	2	2			4	4		
Ovarian hemorrhagic cyst	2			1	1				2		
Ovarian coronal cyst	1		1					1			
Oviduct ovarian cyst	2				2				1	1	
Mesosalpinx cyst	2		1		1			1			1
Ovarian fibroma	1				1				1		
Ovarian adenofibroma	1				1					1	
Ovarian fibrotheca cell tumor	4				4				4		
Chronic ovarian suppurative inflammation	1			1					1		
Hydrosalpinx	1			1					1		
Fallopian tube abscess	1			1					1		
Malignant											
Ovarian serous cystadenocarcinoma	4				2	2				1	3
Ovarian mucinous cystadenocarcinoma	1					1					1
Ovarian endometrioid adenocarcinoma	2					2					2
Ovarian dysgerminoma	2					2					2
Ovarian yolk sac tumor	2					2					2
Ovarian granulosa cell tumor	1					1					1
Ovarian clear cell carcinoma	2				1	1					2
Ovarian carcinosarcoma	1					1					1
Ovarian fibrosarcoma	1				1					1	
Ovarian metastatic carcinoma	4				1	3				1	3
Serous fallopian tube carcinoma	1					1					1
Ovarian borderline serous cystadenoma	5				3	2				3	2
Ovarian borderline mucinous cystadenoma	2				2					1	1
Ovarian borderline serous cystic fibroma	1			1					1		
Total	100		19	29	32	20		19	47	11	23

### Classification of O-RADS combined with CEUS

**Fig. 2:**
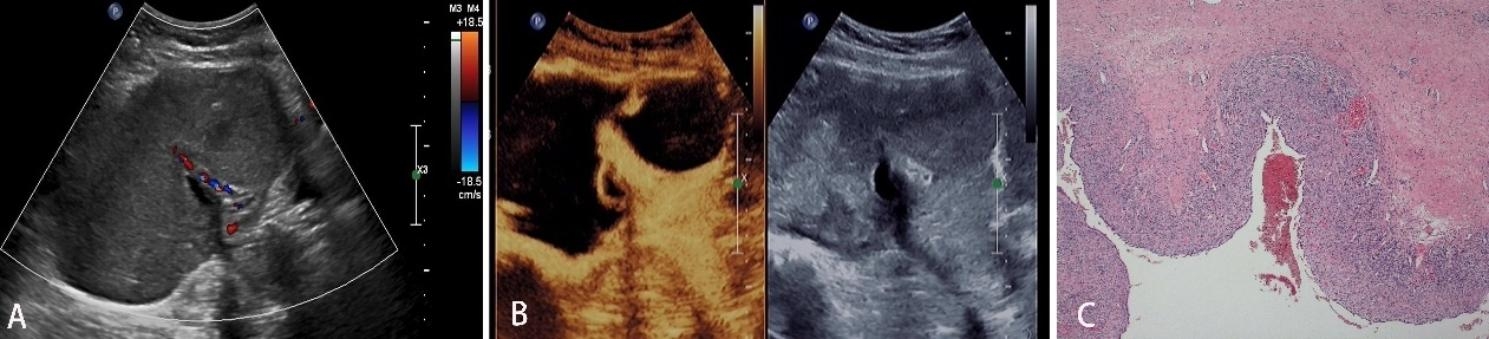
Transabdominal ultrasound and pathological image of a 23-year-old patient with a right adnexal mass. **A:** Conventional ultrasound indicated that the maximum diameter of the mass was 14.2 cm, irregular in shape and solid in appearance; CDFI showed the strip-shaped blood flow signal in the center of the mass, which was classified as 5 by O-RADS. **B:** CEUS 20s showed high annular enhancement at the rim of the mass, and there was no enhancement inside except for the separation, which was downgraded to 3 using O-RADS combined with CEUS. **C:** Pathological results indicated a right ovarian endometriosis cyst (HE × 40)

**Fig. 3:**
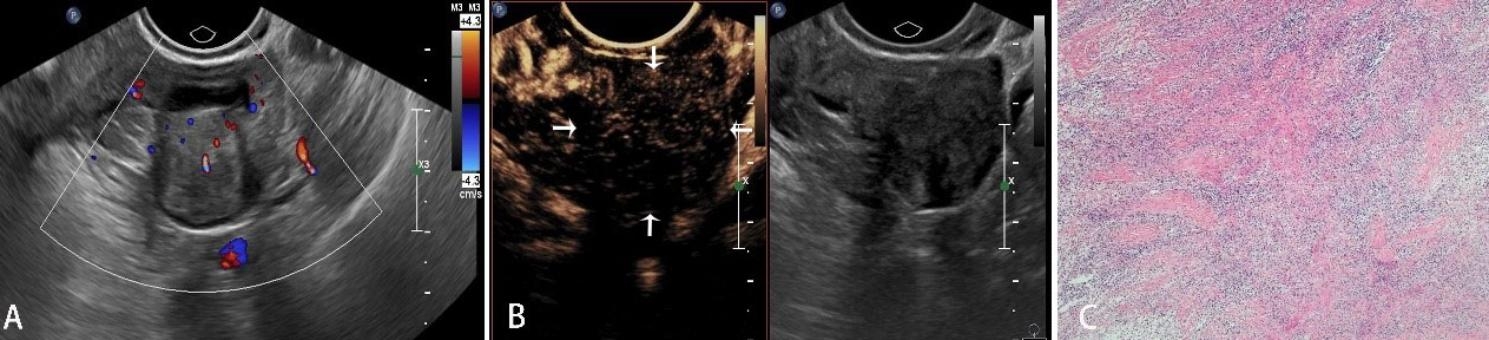
Transvaginal ultrasonogram and pathological image of a left adnexal mass in a 59-year-old patient. **A:** Conventional ultrasound showed that the mass had a maximum diameter of 2.7 cm, regular in shape. CDFI showed the short strip-shaped blood flow signal inside the mass, which was classified as 4 by O-RADS. **B:** CEUS showed hypoenhancement at 38s(arrow); it was downgraded to 3 by O-RADS combined with CEUS. **C:** The pathological results revealed that it was the left ovarian fibrotheca cell tumor (HE × 40)

**Fig. 4:**
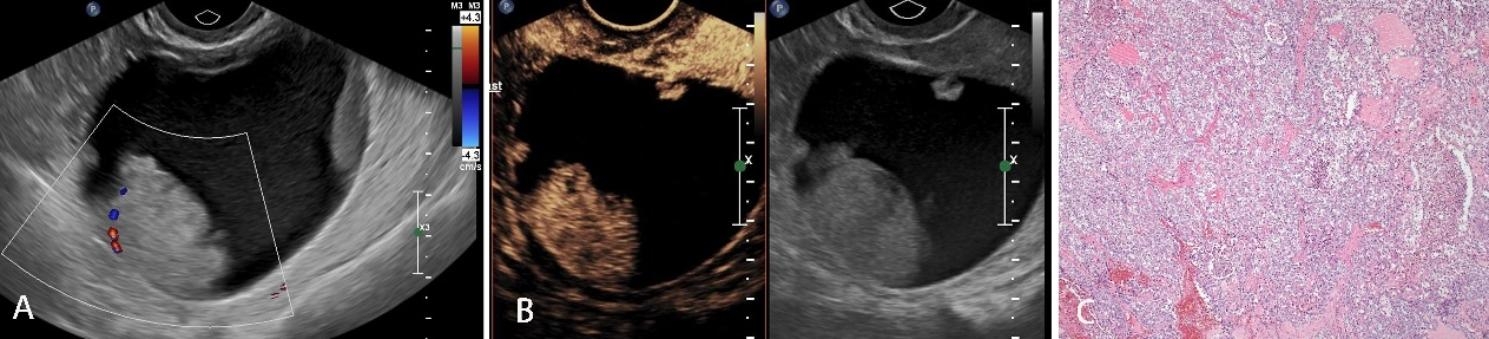
Transvaginal ultrasonogram and pathological image of left adnexal mass in a 50-year-old patient. **A:** Conventional ultrasound showed a regular unilocular cyst with a maximum diameter 8.8 cm, with three solid nodules. CDFI showed a little blood flow signals at the rim of the largest nodule. The mass was classified as 4 by O-RADS. **B:** CEUS showed annular rim enhancement at 33s, and all the internal nodules displayed nonhomogeneous hyperenhancement; then, it was adjusted to 5 by O-RADS combined with CEUS. **C:** Pathological results confirmed it as left ovarian clear cell carcinoma (HE × 40)

As observed by CEUS, there were 51 hyperenhancement, 41 isoenhancement, and 8 hypoenhancement on the edge of the lesions. Moreover, 13 focus vessels were thick, distorted or irregular, and 54 featured edge annular enhancement. Regarding the interior of the lesions, 42 were hyperenhanced, 20 were isoenhanced, 12 were hypoenhanced, and 26 were non-enhanced. There were 23 lesions showing early wash-in, and 77 late or synchronous wash-in. Of them, 62 presented with homogeneous enhancement and 38 inhomogeneous enhancement. After the adjustment using O-RADS combined with CEUS, the adnexal masses were classified as 2, 3, 4, and 5, which were 19(19%),47(47%),11(11%) and 23(23%), respectively. The corresponding proportions of malignancy were 0% (0/0), 2.1% (1/47), 63.6% (7/11) and 91.3% (21/23), respectively. There were 18 benign tumors which were downgraded (Figs. 2 and 3), and  3 malignant tumors were upgraded (Fig. 4).

### Diagnostic performance of O-RADS and O-RADS combined with CEUS in adnexal masses

**Fig. 5:**
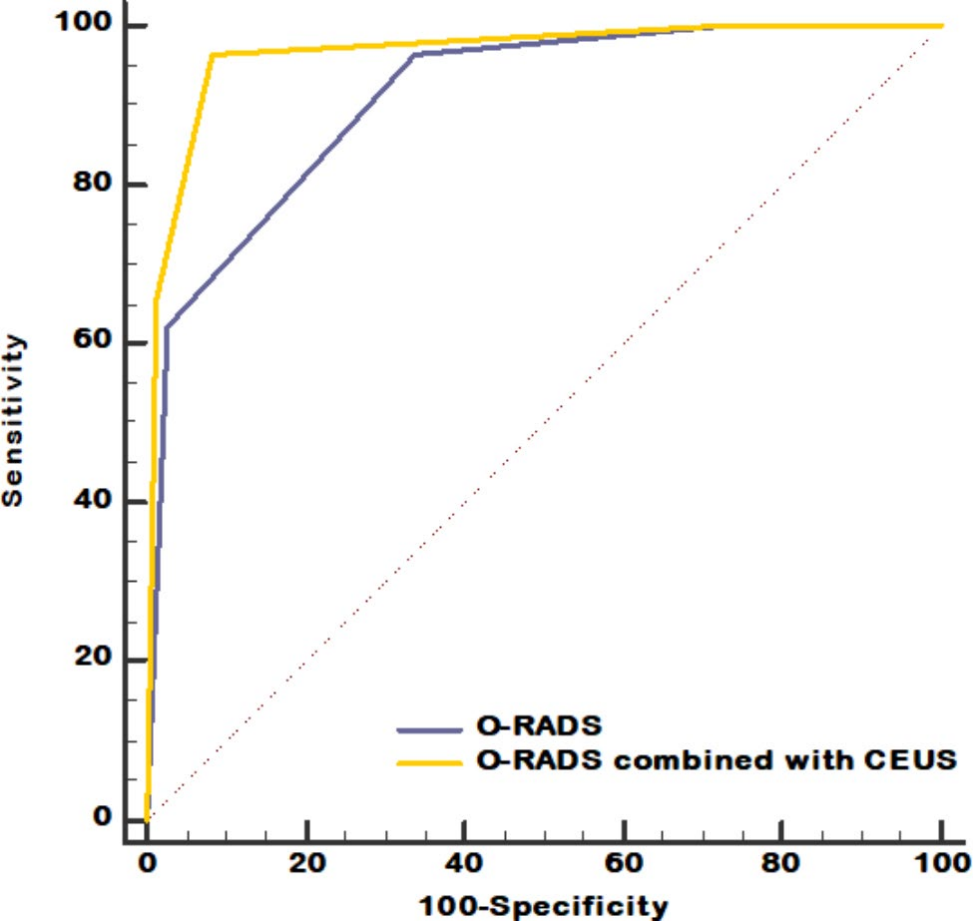
AUC of the ROC curves of the two methods in the discrimination of malignant from benign adnexal masses

Taking O-RADS > 3 as the cut-off value for the diagnosis of adnexal malignancy, the AUC was 0.910, and using O-RADS combined with CEUS > 3 as the cut-off value, the AUC was 0.962 (Fig. 5). The sensitivity, specificity, accuracy, PPV, and NPV of both are shown in Table 2. The specificity, accuracy, PPV, and AUC of O-RADS combined with CEUS were significantly higher than those of O-RADS (*P* < 0.01). Both methods had excellent sensitivity and NPV but there were no significant differences between them(*P*＞0.05).

**Table 2:** Comparison of diagnostic efficiency between the two methods

Diagnostic method	Sensitivity (%)	Specificity (%)	Accuracy (%)	PPV(%)	NPV(%)	AUC
O-RADS	96.6	66.2	75.0	53.8	97.9	0.910
O-RADS combined with CEUS	96.6	91.5	93.0	82.4	98.5	0.962
χ^2^ /Z	＜0.001	13.693	12.054	7.355	1.000	2.689
*P*	1.000	＜0.001	0.001	0.007	0.667	0.007

## Discussion

The features of multiple pathological types and diverse ultrasound images in adnexal tumors, and the existence of overlapping images of both benign and malignant tumors, render the clinical diagnosis challenging. The O-RADS guidelines help standardize ultrasonic risk assessment and improve the accuracy of benign and malignant judgment of adnexal tumors [7, 9].

Studies have shown that O-RADS is significantly more sensitive to malignant tumors than GI-RAD and IOTA [10, 15]. Taking O-RADS > 3 as the cut-off value for malignant tumors, the sensitivity and NPV were 96.6% and 97.9%, respectively, while the specificity and PPV (66.2% and 53.8%) were relatively low. The results were comparable to those of Hack et al. [9]. In this study, 96.6% (28/29) of the benign tumors in O-RADS 3 and 90% (18/20) of the malignant tumors in O-RADS 5 exhibited excellent specificity. However, the ratio of benign to malignant tumors in O-RADS 4 was almost 2:1, which means that it was tricky to identify benign and malignant masses in O-RADS 4, hence the main reason for the low specificity of O-RADS. Due to the limitations of conventional ultrasound on low-speed blood flow and covering some solid-like components, it is thorny to determine whether some lesions have active components through color Doppler flow image(CDFI) like atypical teratoma, endometriosis cyst with clot and separation, hemosiderin particle deposition, etc. Furthermore, some regular solid lesions such as ovarian fibrotheca cell tumors accompanied by blood flow signals may be upgraded the O-RADS classification, resulting in a high proportion of O-RADS 4 and an increase in false positivity, this point was also confirmed by Lu et al. [16].

CEUS technology can clearly display small vessels (vascular caliber less than 200 μm) and detect the low-speed blood flow of 0.1–10 mm/s in the microvessels through the nonlinear effect of contrast agent in the sound field and the principle of the strong backscattered signal [17]. It overcomes the limitations of routine ultrasonic CDFI and considerably improves the judgment of whether solid-like components of the mass have a blood supply. In this study, twelve cystic-solid masses in O-RADS 4 were observed, while they were downgraded to O-RADS 3 since the “solid components” detected by conventional ultrasound showed no blood supply confirmed using CEUS. Subsequently, pathological findings confirmed that most of them were endometriosis cysts, hemorrhagic cysts, and mucinous cystadenomas. It is illustrated that CEUS significantly improves the evaluation accuracy.

There are differences in angiogenesis and blood perfusion patterns between benign and malignant tumors. Some scholars thought that the morphology of blood vessels can be used to predict benign and malignant tumors [18]. Hence, the color blood flow score is considered as an important indicator of O-RADS guidelines for assessing high-risk lesions. Studies [11, 19, 20] have shown that CEUS in malignant ovarian tumors frequently showed thick and distorted blood vessels, early wash-in, and internal inhomogeneous hyperenhancement. In contrast, benign tumors frequently showed envelope annular enhancement, late wash-in, and interior hypoenhancement or non-enhancement. The present study exhibited similar results except for wash-in. Accordingly, the presence of malignant angiographic signs in O-RADS 2 and 3 masses indicates that there are solid components or disturbance of tumor vascularity, so the possibility of malignancy could not be excluded, and the classification should be upgraded. In addition, O-RADS 4 and 5 masses with benign angiographic features, particularly edge ring-enhancement with interior non-enhancement, strongly suggest a higher possibility of benign lesions; therefore, the classification should be downgraded. The efficiency of the combination of O-RADS with CEUS was significantly higher than that of O-RADS alone. However, some lesions were not easy to assess even using the combined method. For example, there was one case of ovarian goiter, which was classified as 5 using both O-RADS and O-RADS combined with CEUS; and another case of ovarian borderline serous cystic fibroma was classified as 3 using the two methods. It demonstrates that the morphology and blood perfusion patterns of a few benign and malignant tumors overlap.

Compared to O-RADS, the means of O-RADS combined with CEUS improved the diagnostic specificity and PPV, and remained a high sensitivity and NPV. The limitations of this study are: (1) Although the use of CEUS in differentiation adnexal masses has been studied to a certain extent, there is no unified diagnostic standard, and the design method of this study might be subjective to a certain degree; (2) The enrolled cases were all surgical cases, which might have selection bias; (3) It was a single-center small-sample study with too few cases of malignant tumors, and the research results need to be further verified by a multiple-center study with a larger sample size.

## Conclusion

The study revealed that combination of O-RADS and CEUS has more excellent performance in differentiation of benign and malignant adnexal masses and it may be a promising application value in the risk stratification of adnexal tumors through promoting the accuracy of final assessment categories.

## Data Availability

The data supporting the current study are available from the corresponding author upon reasonable request.
